# Orthopedia Transcription Factor *otpa* and *otpb* Paralogous Genes Function during Dopaminergic and Neuroendocrine Cell Specification in Larval Zebrafish

**DOI:** 10.1371/journal.pone.0075002

**Published:** 2013-09-20

**Authors:** António M. Fernandes, Erin Beddows, Alida Filippi, Wolfgang Driever

**Affiliations:** Developmental Biology Unit, Faculty of Biology, and BIOSS Centre for Biological Signalling Studies, University of Freiburg, Freiburg, Germany; University Zürich, Switzerland

## Abstract

The homeodomain transcription factor Orthopedia (Otp) is an important regulator for specification of defined subsets of neuroendocrine cells and dopaminergic neurons in vertebrates. In zebrafish, two paralogous *otp* genes, *otpa* and *otpb*, are present in the genome. Neither complete loss of Otp activity nor differential contributions of Otpa and Otpb to specification of defined neuronal populations have been analyzed in detail. We characterized zebrafish embryos and early larvae mutant for null alleles of *otpa, otpb*, or both genes to determine their individual contributions to the specification of *th* expressing dopaminergic neuronal populations as well as of *crh, oxt, avp, trh* or *sst1.1* expressing neuroendocrine cells. *otpa* mutant larvae show an almost complete reduction of ventral diencephalic dopaminergic neurons, as reported previously. A small reduction in the number of *trh* cells in the preoptic region is detectable in *otpa* mutants, but no significant loss of *crh*, *oxt* and *avp* preoptic neuroendocrine cells. *otpb* single mutant larvae do not display a reduction in dopaminergic neurons or neuroendocrine cells in the *otp* expressing regions. In contrast, in *otpa* and *otpb* double mutant larvae specific groups of dopaminergic neurons as well as of *crh, oxt, avp, trh* and *sst1.1*-expressing neuroendocrine cells are completely lost. These observations suggest that the requirement for *otpa* and *otpb* function during development of the larval diencephalon is partially redundant. During evolutionary diversification of the paralogous *otp* genes, *otpa* maintained the prominent role in ventral diencephalic dopaminergic and neuroendocrine cell specification and is capable of partially compensating *otpb* loss of function. In addition, we identified a role of Otp in the development of a domain of *somatostatin1*-expressing cells in the rostral hindbrain, a region with strong *otp* expression but so far uncharacterized Otp function. Otp may thus be crucial for defined neuronal cell types also in the hindbrain.

## Introduction

Patterning and neuronal differentiation in the vertebrate brain are controlled by a diverse group of transcription factors highly conserved throughout evolution. The Orthopedia homeodomain transcription factor encoding genes were initially discovered in both *Drosophila* and mouse based on their homeobox sequence, and characterized for their expression in the central nervous system [1]. Analysis of *Otp* mutant mice, which die shortly after birth, revealed that Otp contributes to patterning in the hypothalamus and preoptic region, and is required for differentiation of specific oxytocin (OT), arginine vasopressin (AVT), corticotropin-releasing hormone (CRH) and somatostatin (SS) expressing cells in the paraventricular, supraoptic, anterior periventricular, and arcuate nuclei [2], [3]. It was further revealed that *Otp* acts in parallel with the transcription factor *Sim1* and both of them are required to maintain *Brn2* expression for terminal differentiation of neurosecretory cells in the mouse hypothalamus [3]. *Otp* expression in the hypothalamus was shown to be highly conserved across tetrapods [4]. Further, already in ascidian embryos an *otp* gene is expressed in the hypothalamus adjacent to the sensory vesicle [5], which may derive from a proto-neuroendocrine territory in a chordate ancestor [6]. In humans, OTP is also expressed in the hypothalamus [7]. The expression of *otp* in the preoptic region (PO) is also highly conserved in chordates [4].

Work in zebrafish revealed that Otp is also required for the development of a specific subset of dopaminergic (DA) neurons in the hypothalamus and posterior tuberculum in zebrafish and of the homologous A11 group DA neurons in the dorsal hypothalamus of mice [8]. Based on their relevance to human diseases, including Parkinson’s and schizophrenia, intensive research efforts have been focused on ventral midbrain DA neurons [9]–[11]. In contrast, DA neurons in other parts of the brain [12], specifically the ventral diencephalon, have received relatively little attention. The small size and transparent nature of the larvae in combination with the genetics have made zebrafish a good system to investigate DA development [12]–[16]. In zebrafish, the Otp-dependent DA neurons are of particular interest because they represent the most prominent far projecting DA system in larval zebrafish [17], and are the only DA group sending projections ascending to the telencephalon, descending to hindbrain and spinal cord, as well as contributing to endohypothalamic circuitry [18]. Work in mammals showed that the Otp-dependent A11 DA group appears to have a projection pattern very similar to zebrafish [19]. In zebrafish, mutations in only one of the two paralogous *otpa* and *otpb* genes have been previously reported [8]. o*tpa* mutants show a reduction in four posterior tubercular and hypothalamic DA groups, termed DC 2, 4, 5 and 6 based on the nomenclature proposed by Rink and Wullimann [20], but not a total loss of these DA neurons [8].

While the contribution of Otp to neuroendocrine development has been studied in detail in mice [2], [3], this function and potentially evolutionary conserved aspects are less well understood in the zebrafish system. As in mammals, two major types of neuroendocrine systems can be distinguished in the hypothalamus in zebrafish [21], the parvocellular and the magnocellular systems. Parvocellular neuroendocrine cells send projections to the adenohypophysis (anterior lobe of pituitary) and release several peptides, which include thyrotropin-releasing hormone (TRH), corticotropin-releasing hormone (CRH) and somatostatin (SST). Magnocellular neurons synthesize oxytocin (*oxt*; previously termed isotocin neurophysin *itnp* [22]) and arginine vasopressin- like (*avp*; also termed vasotocin neurophysin *vsnp* [23]) and project to the neurohypophysis (posterior lobe of the pituitary). Similar to mammals, in fish *otp* together with *arnt2* and its binding partner *sim1* were shown to be a core component of a conserved transcriptional network for neuroendocrine cells [24]. The expression of *otp* in the preoptic region is highly conserved in tetrapods and is assumed to be plesiomorphic among chordates [4]. In zebrafish, it was reported that *otp* is necessary for *oxt*- and *avp*-producing cells in the PO [23], [25]. In a recent study, a contribution of Otp activity to development of caudal hypothalamic Vasoactive intestinal peptide hormone secreting cells was also shown [26].

Here, we investigated in detail the contributions of the paralogous *otpa* and *otpb* genes to neuroendocrine development in zebrafish. We found that both genes act in a partially redundant manner regarding DA neuron and neuroendocrine cell specification. While *Otpb* appears dispensable for DA neuron and neuroendocrine cells specification in the presence of *otpa*, loss of *otpb* enhances the *otpa* mutant phenotype, resulting in the complete loss of specific neuronal groups. Surprisingly, we found that *otp* genes are also important for the development of a group of *somatostatin1.1*-expressing cells in the hindbrain. *Otp* is strongly expressed in the hindbrain in both mammals and zebrafish [1]–[3], [8], but its requirement during development of specific neurons in the hindbrain has not previously been reported.

## Materials and Methods

### Zebrafish Husbandry

Zebrafish breeding and maintenance were carried out under standard conditions at 28.5°C [27]. Experiments were performed with *otpa^m866^* [8] and *otpb^sa115^* (obtained from Sanger Zebrafish Mutation Project) mutant alleles. To inhibit pigmentation, embryos were incubated in egg water containing 0.2 mM 1-phenyl-2-thiourea. All the experimental procedures were in accordance with the German laws for animal care.

### Genotyping

The genotype of *otp* alleles was determined by genomic PCR using dCAPS assays [28]. The *otpa^m866^* mutant fish were genotyped using the following primer pair: otpa-m866-F1 5′-ggtcacagggaggcattaaa-3′ and otpa-m866-R1 5′-gatagtgggttttggcgaag-3′. The 310 bp PCR product was then digested with Hpy188III. Upon restriction, the wild type allele results in two fragments of 170 bp and 140 bp. The *m866* mutation abolishes the restriction site, therefore the mutant allele is not cut by restriction with Hpy188III. For genotyping of *otpb^sa115^* mutants the following primer pair was used: otpb-sa115-F1 5′-aggtcaacgccaaagaccaa-3′ and otpb-sa115-R1 5′-gcgatcggaaacatatttga-3′. The 399 bp PCR product was then digested with BbvI. Restriction results in two fragments (374 bp and a 25 bp) for the wild type allele and in one uncut fragment for the mutant allele.

### In situ Hybridization

Larvae were fixed in 4% paraformaldehyde in phosphate-buffered saline at three days post-fertilization. Standard colorimetric whole-mount in situ hybridization (WISH) and fluorescent WISH were performed as previously described [29]. The following digoxigenin-labeled riboprobes were synthesized: *th* [13], *crh, trh, sst1.1, oxt/itnp* [24], *avp*/*vsnp* [30] and *gad2/gad65* and *gad1b/gad67* [31]. A mixture of antisense digoxigenin-labeled riboprobes against *vglut2a/slc17a6b* and *vglut2b/slc17a6a* [32] were used to detect glutamatergic neurons and a *glyt2/slc6a5* probe [32] for glycinergic neurons.

### Sequence Alignments

Otp protein sequences were aligned and analyzed with Clustal X2 [33] and CLC Genomics Workbench 5 (http://www.clcbio.com).

### Microscopy, Cell Quantification and Image Analysis

Transmitted light images were acquired using a Zeiss Axioskop compound microscope. For quantification of cell numbers from WISH experiments (Figure S2), an Axio Examiner.D1 microscope (Carl Zeiss) with the transmitted-light differential interference contrast (DIC) illumination technique and a high numerical aperture 20× NA 1.0 lens was used to count cells at single-cell resolution in WISH stained embryos (Figure S3). The high numerical aperture lens enabled optical sectioning to obtain cellular resolution even when cells were densely packed in clusters. With DIC illumination best images were acquired with the iris diaphragm completely open. A Z-stack (1 µm steps) of images was recorded. Zen software was used to mark and count WISH stained cells in each stack. NIH ImageJ software and Adobe Photoshop were used to compose figures. DOG 1.0 [34] and Inkscape [www.inkscape.org] software were used for schematic drawings.

### Statistical Analysis

Cell numbers from the different genotypes analyzed were compared with wildtype larvae using the Wilcoxon–Mann–Whitney rank-sum non-parametric test. Statistical analysis were performed with the help of the Excel add-in MegaStat (http://glencoe.mcgraw-hill.com/sites/0010126585/student_view0/megastat.html).

## Results

### *otpb^sa115^* Mutant Otpb Protein Lacks the Highly Conserved Homeodomain

**Figure 1:**
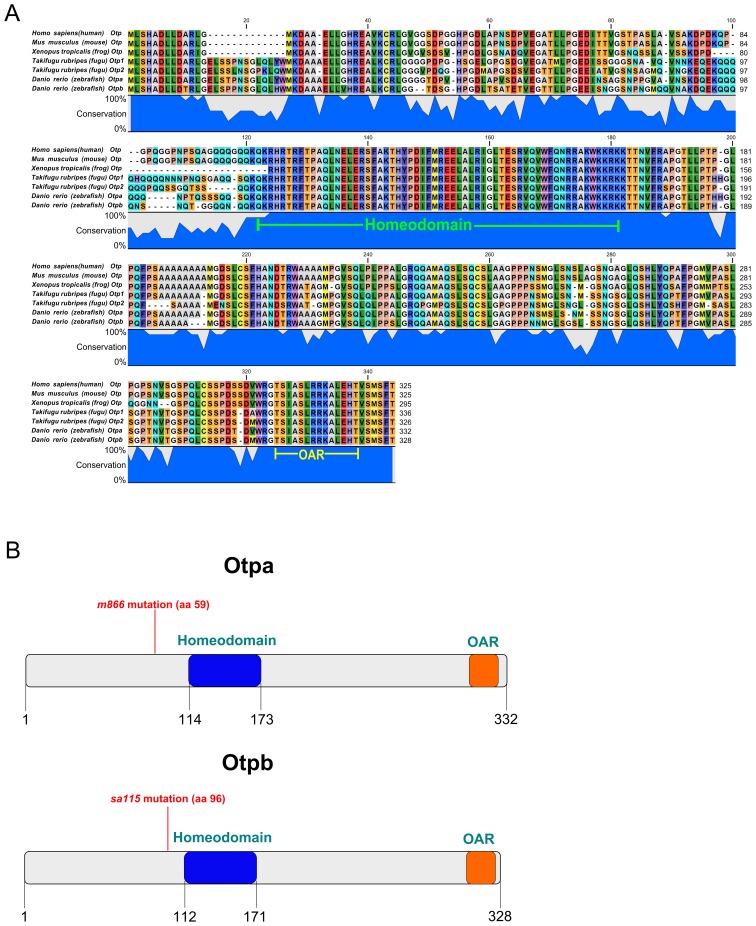
*Otpb^sa0115^* mutants lack the highly conserved Homeodomain. (A) The Otp protein and specifically the homeodomain sequence (green label) are highly conserved in vertebrates. The conserved OAR domain present in all Otp proteins in vertebrates is also depicted (yellow label). (B) Schematic representation showing both Otpa and Otpb protein structure and position of stop codons caused by mutations. In the *otpa^m866^* allele the mutation results in a frameshift and a premature stop codon after additional 59 amino acids. In the *otpb^sa0115^* allele a base-pair deletion results in a premature stop codon 96 amino acids downstream of the start codon. Both mutations generate smaller proteins which completely lack the highly conserved homeodomain (in blue). The conserved OAR (otp, aristaless, rax), potential interaction domain present in several paired-like homeodomain proteins and all Otp proteins in vertebrates is also depicted (orange).

The Otp homeodomain is extremely well conserved from fish to human, and both zebrafish Otpa and Otpb proteins share this high conservation among vertebrates (Figure1 A). In addition, more carboxyterminal regions are highly conserved, including the OAR (otp, aristaless, rax) potential interaction domain present in several paired-like homeodomain proteins. While mutations in the *otpa* locus have been previously characterized [8], a mutant allele for the paralogous gene *otpb* has not been reported so far, and information concerning the phenotype of complete loss of Otp function is limited to Morpholino knockdown studies [8], [35]. Recently, the potential null allele *otpb^sa115^* was made available by the Sanger Zebrafish Mutation Project. The *otpb^sa115^* allele carries a base-pair deletion at amino acid 96 of the *otpb* ORF, which causes a premature stop codon before the highly conserved homeodomain (Figure 1B, bottom). This *otpb* mutation likely results in the production of a short, nonfunctional protein lacking the entire Otp homeodomain. Therefore, similar to *otpa^m866^, otpb^sa115^* is likely a null allele with complete loss of function (Figure 1B, top).

### *otpa* and *otpb* are Differentially Required for the Development of Dopaminergic Neurons in the Ventral Diencephalon

**Figure 2:**
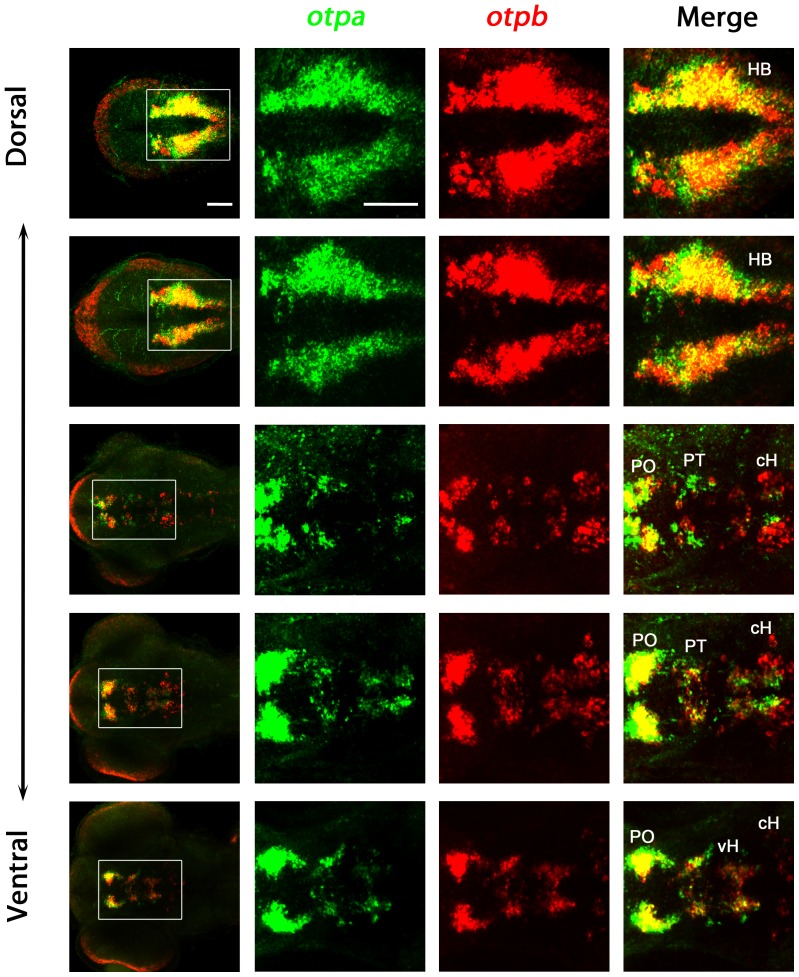
Expression of *otpa* and *otpb* in wildtype larvae. Expression of *otpa* and *otpb* were detected by double fluorescent whole mount in situ hybridization of wildtype larvae fixed at 3 dpf. From the whole confocal image stack, sub-stacks ranging from dorsal hindbrain image planes to ventral forebrain planes were used to generate a series of dorso-ventral Z-projections. The data reveal that *otpa* and *otpb* have overlapping expression but also non-overlapping domains. Dorsal view, anterior at left. Abbreviations: cH, caudal hypothalamus; HB, hindbrain; PO, preoptic region; PT, posterior tuberculum; vH, ventral hypothalamus. Scale bar is 50 µm.

Although *otpa* and *otpb* expression domains show a high degree of overlap (e.g. in the preoptic region and hindbrain; Figure 2), there are regions where just one of the *otp* genes is expressed (e.g. *otpa* in the medial periventricular area of the caudal hypothalamus, *otpb* in the more lateral caudal hypothalamus; Figure 2). This suggests that specific neuronal populations may differentially rely on *otpa* or *otpb* activity. To investigate the contributions of *otpa* and *otpb* to development of zebrafish DA neurons, we analyzed *otpa* and *otpb* homozygous single mutants and generated *otpa;otpb* double mutant embryos. While *otpa* homozygous as well as *otpb* homozygous fish are adult viable, *otpa;otpb* double mutant embryos and larvae develop morphologically normal and may form swim bladders, but die during late larval stages before juvenile ages. Due to the lethality at larval stages, the function of both *otp* genes for the specification of specific neuronal populations in adult zebrafish could not be addressed using double mutants.

**Figure 3:**
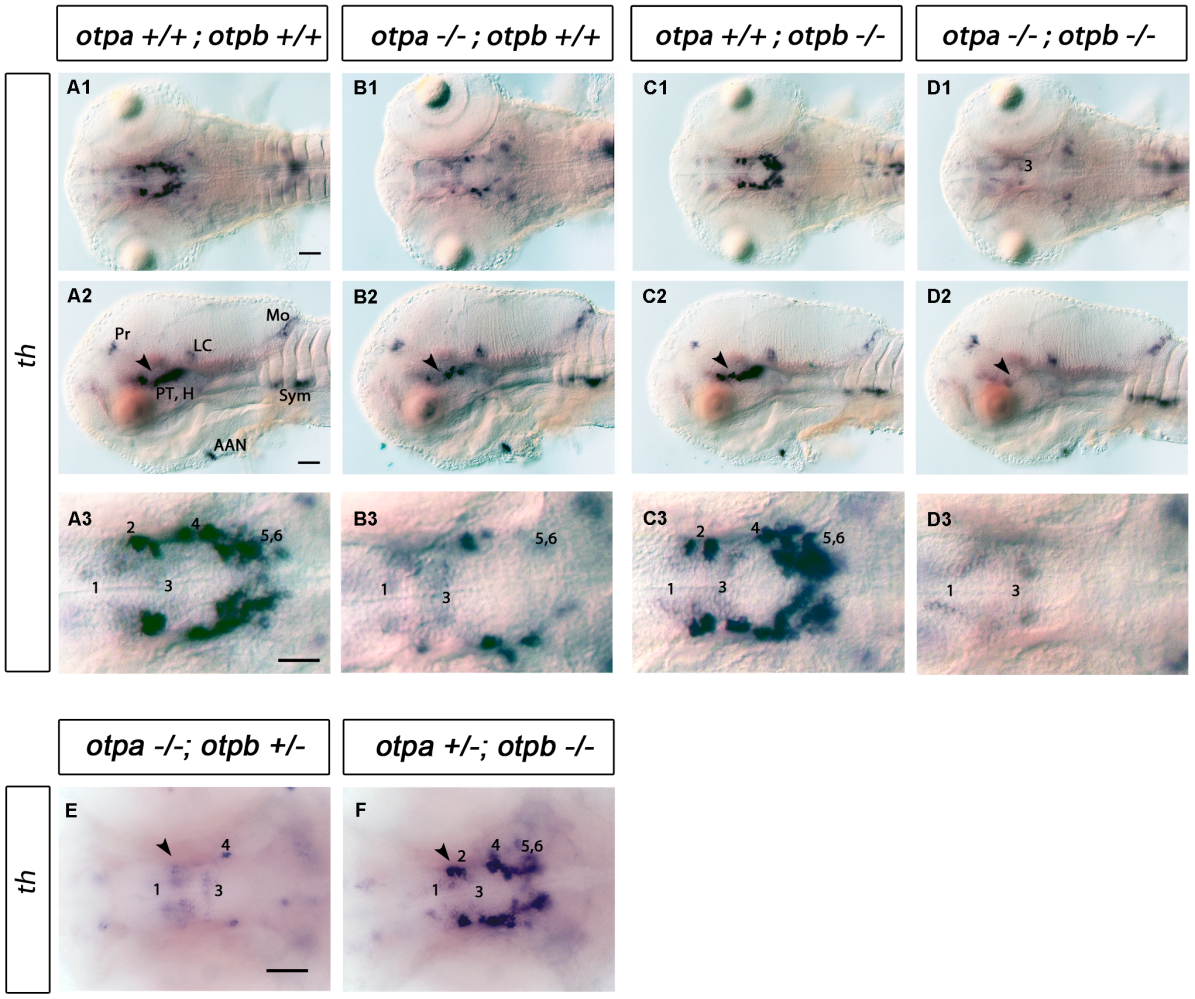
Analysis of DA neurons by expression of *th* in *otpa* and *otpb* single and double mutant larvae. (A–D) Whole-mount in situ hybridization of 3 dpf larvae reveals reduction of *th* expression in the posterior tuberculum of *otpa* and total loss of the expression in *otpa;otpb* double mutants (arrowhead). Other *th* expressing domains are not affected. (A1–D1, A3–D3) Dorsal views, anterior at left; (A2–D2) lateral views, dorsal up. Scale bar is 50 µm. (E,F) Whole-mount in situ hybridization of 3 dpf larvae reveals reduction of *th* expression in the posterior tuberculum of *otpa* mutant, *otpb* heterozygous larvae (E) (arrowhead). No clear reduction is detected in the posterior tuberculum of *otpb* mutant, *otpa* heterozygous larvae (F) (arrowhead). Dorsal view, anterior at left. Scale bar is 50 µm. Abbreviations: AAC, arch associated cluster; DC, diencephalic cluster; H, hypothalamus; LC, locus coeruleus; MO, medulla oblongata; Pr, pretectum; PT, posterior tuberculum. Numbers indicate dopaminergic neurons in the ventral thalamic cluster (1) and posterior tuberculum/hypothalamus (2–6) according to [20]. Scale bar is 50 µm.

*otpb^sa115−/−^* mutant larvae develop DA neurons in normal numbers and anatomical positions, indistinguishable from wildtype siblings (compare Figure 3 A1–3 with C1–3). This is in contrast to the clear reduction of specific DA neurons in *otpa* mutants (Figure 3 B1–3) [8].

Complete loss of Otp activity in *otpa*;*otpb* double mutants caused a more severe phenotype than *otpa* mutants alone. The double mutants displayed a complete loss of DA groups 2, 4, 5 and 6 in the ventral diencephalon (Figure 3 D1–3). We also noticed that fish homozygous mutant for *otpa* and heterozygous for *otpb* show a stronger reduction of DA neurons in the posterior tuberculum (Figure 3E), while larvae homozygous mutant for *otpb* and heterozygous for *otpa* showed no evident phenotype (Figure 3F). This suggests that most of the Otp activity required for DA differentiation in the PT/vDC is provided by *otpa*, while *otpb* activity makes a minor, albeit significant contribution.

### *otpa* and *otpb* Control Development of *crh* Expressing Cells in Preoptic and Ventral Diencephalic Regions

**Figure 4:**
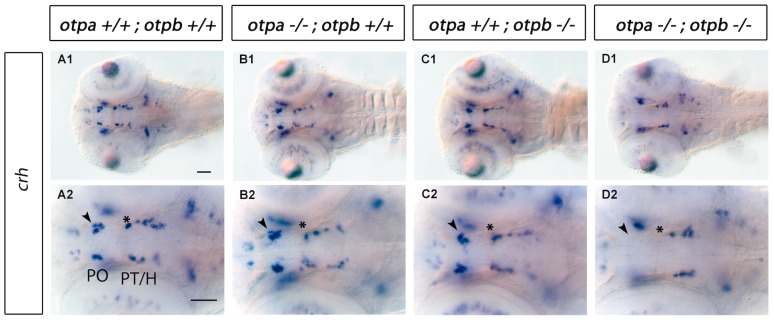
Expression of *crh* in *otpa* and *otpb* single and double mutant larvae. Whole-mount in situ hybridization of 3 dpf larvae reveals loss of *crh* expression in the preoptic region and posterior tuberculum of *otpa;otpb* double mutant larvae (arrowhead and asterisk, respectively). Dorsal view, anterior at left. Scale bar is 50 µm. Abbreviations: H, hypothalamus; PO, preoptic region; PT, posterior tuberculum.

Corticotropin-releasing hormone (CRH) has been described as being secreted by the paraventricular nucleus (PVN) of the hypothalamus in response to stress [36]–[38]. *crh* is expressed in several regions of the embryonic zebrafish brain, including telencephalon, hypothalamus, posterior tuberculum, thalamus, retina and hindbrain [39]. *crh* positive neurons in the posterior tuberculum and hypothalamus were shown to be intermingled with DA neurons of the ventral diencephalic groups [24], [39]. In *otpa* or *otpb* single mutants we did not detect any significant changes in number or location of *crh*-expressing cells compared to wildtype siblings (Figure 4A–C, for cell counts see Figure S2E). In contrast, *otpa;otpb* double mutants showed a complete loss of *crh*-expressing cells in defined *crh* neuronal clusters in the PO region (Figure 4D1,D2 arrowhead) and a clear reduction of *crh*-expressing cells in the most anterior PT domain (Figure 4D1,D2 asterisk, for cell counts see Figure S2F). This suggests that both *otpa* and *otpb* genes act functionally redundant during specification of *crh*-expressing neurons in zebrafish (see also Figure S1G,H). Interestingly, only a defined subset of *crh* neuronal groups depends on Otp activity, while others, including the more caudal hypothalamic *crh* neurons, are apparently specified by Otp-independent mechanisms.

### *otpa* and *otpb* are Required for Development of *oxt, avp* and *trh* Neurons in the Preoptic Region

Oxytocin (*oxt,* in fish previously named isotocin-neurophysin *itnp*) and arginine vasopressin (*avp,* previously *vsnp*) influence several behavioral and physiological processes such as reproductive, maternal, and aggression behaviors, as well as learning and memory [40]–[42]. Thyrotropin-releasing hormone (*trh*) has several important roles including regulation of energy homeostasis, feeding behavior and locomotion activation [43].

**Figure 5:**
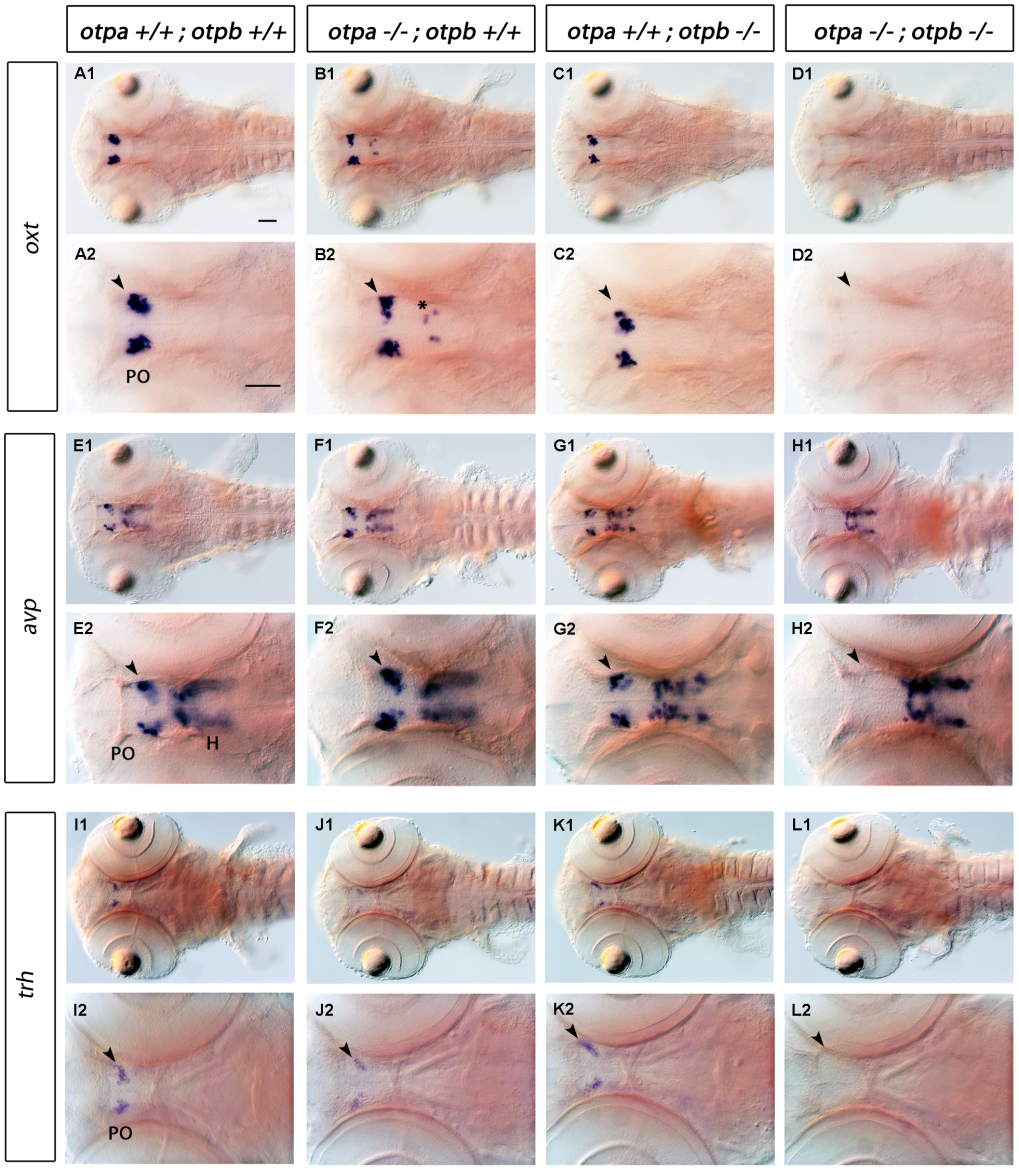
Expression of *oxt, avp and trh* in *otpa* and *otpb* single and double mutant larvae. Whole-mount in situ hybridization reveals loss of *oxt, avp and trh* expression in the preoptic region (arrowhead) of *otpa;otpb* double mutant larvae at 3 dpf. We detected *oxt*-expressing cells at ectopic locations within the diencephalon in *otpa* mutants (B2, asterisk). A reduction of *trh*-expressing cells in the preoptic region in *otpa* single mutants is detectable, (J2, arrowhead). Dorsal view, anterior at left. Scale bar is 50 µm. Abbreviations: H, hypothalamus; PO, preoptic region.

Based on *otpb* Morpholino knockdown it was previously postulated that *otpb* is necessary for *oxt*- and *avp*-producing cells in the PO [23], [25]. In contrast to these reports, we did not observe a significant reduction in cells expressing *oxt* and *avp* in *otpb^sa0115^* mutants (Figure 5 A, C, E, G, for cell counts see Figure S2A and C). We also did not observe a phenotype affecting *trh* expressing cells in *otpb^sa0115^* mutants (Figure 5 K, compare with I, for cell counts see Figure S2D). Moreover, in *otpa^m866^* single mutants we did not detect a clear reduction in *oxt* and *avp* expressing cells in the PO region (Figure 5 B, F). However, we detected *oxt*-expressing cells at ectopic locations within the diencephalon in *otpa* mutants (Figure 5 B2, asterisk, for cell counts see Figure S2B). The number of cells expressing *trh* in the PO region was significantly reduced in *otpa^m866^* mutants when compared to wild-type or the *otpb* mutant (Figure 5 J2, arrowhead, for cell counts see Figure S2D). When analyzing the expression of *oxt*, *avp* and *trh* in *otpa;otpb* double mutants, we observed a complete loss of these cell types in the PO region (Figure 5 D, H, L, arrowheads). The analysis of *otpa−/−;otpb+/−* and *otpa+/−;otpb−/−* mutants revealed that for all three neuronal types, *otpa* has a more prominent contribution to neuronal specification than *otpb*, because in each case the *otpa−/−;otpb+/−* phenotype was stronger (Figure S1A-F and Figure S2). In summary, these data reveal a crucial activity of Otp in *oxt*, *avp* and *trh* cell specification and show the partially redundant nature of *otpa* and *otpb* activity.

### *otpa* and *otpb* are Required for the Development of Hindbrain *somatostatin1.1-*expressing Cells

**Figure 6:**
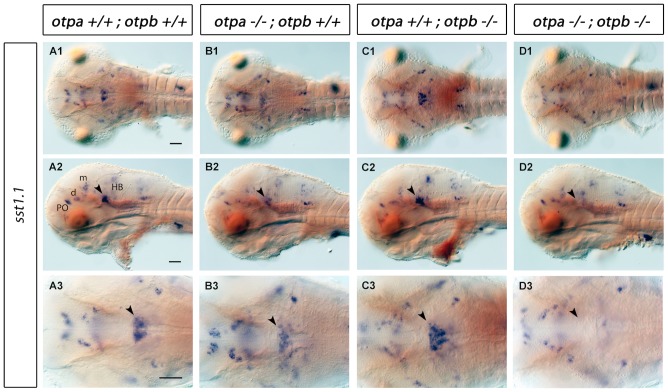
Expression of *sst1.1* in *otpa* and *otpb* single and double mutant larvae. Whole-mount in situ hybridization reveals reduction of *sst1.1* expression (arrowhead) in the rostral hindbrain of *otpa* mutants and total loss of the expression in *otpa;otpb* double mutant larvae at 3 dpf. (A1–D1, A3–D3) Dorsal view, anterior at left; (A2–D2) lateral view, dorsal up. Scale bar is 50 µm. Abbreviations: d, diencephalon; HB, hindbrain; m, mesencephalon; PO, preoptic region.

Somatostatins play important roles for negative regulation of endocrine secretion and regulation of growth in vertebrates. Most notably they also act as neuromodulators in the central nervous system, mediating motor, cognitive and sensory effects [44], [45]. *somatostatin1.1 (sst1.1)* expression in the brain was previously characterized in zebrafish [46.]. In both *arnt2^m1055^* mutants and *sim1a* morphants, *sst1.1* expression was shown to be reduced in the PO region [24]. Surprisingly, we did not observe a strong reduction of *sst1.1* expression in the PO region in *otpa* and *otpb* single or double mutants (Figure 6). However, *sst1.1* expression was reduced in the rostral hindbrain of *otpa* mutant larvae (Fig. 6 B1–B3, arrowhead, for cell counts see Figure S2G). This *sst1.1* expression domain was not affected in *otpb* mutants (Figure 6 C1–C3), whereas in *otpa;otpb* double mutants it was completely lost (Figure 6 D1–D3, arrowhead). Other *sst1.1-*expressing domains appear not to rely on Otp function.

**Figure 7:**
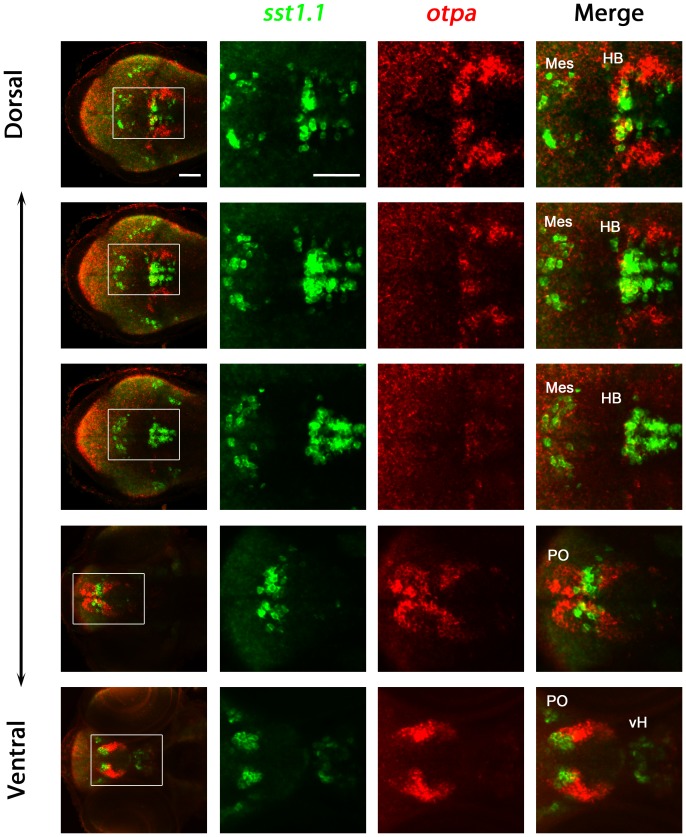
Analysis of coexpression of *otpa* and *sst1.1* in wildtype larvae. Expression of *otpa* and *sst1.1* were detected by double fluorescent whole mount in situ hybridization of wildtype larvae fixed at 3 dpf. From the whole confocal image stack, sub-stacks ranging from dorsal hindbrain image planes to ventral forebrain planes were used to generate a series of dorso-ventral Z-projections. The data reveal that *sst1.1* and *otpa* expression domains overlap in the rostral hindbrain in wildtype larvae at 3 dpf, and some cells appear to coexpress both genes. Dorsal view, anterior at left. Abbreviations: cH, caudal hypothalamus; HB, hindbrain; Mes, mesencephalon; PO, preoptic region; vH, ventral hypothalamus. Scale bar is 50 µm.

The results suggest that *sst1.1* and *otpa* may be coexpressed in some domains but not in others. Therefore, we performed double fluorescent whole-mount *in situ* hybridization for *sst1.1* and *otpa* and compare results between the hindbrain and the preoptic region domains (Figure 7). *sst1.1* and *otpa* coexpression analysis revealed that cells expressing *sst1.1* and *otpa* in the hindbrain are intermingled and some may coexpress both genes, while we could not observe any coexpression of *sst1.1* and *otpa* in cells of the preoptic region at 3 day post fertilization (Figure 7). Analysis of *sst1.1* expression and *otpa* mutants together suggest that *sst1.1* expression may only be affected in areas of coexpression with *otpa*. However, we cannot exclude that *sst1.1* and *otpa* may be coexpressed in other domains at different developmental stages, not analyzed in this study.

### *otpa* and *otpb* are not Required for the Development of Hindbrain Gabaergic, Glycinergic, and Glutamatergic Neurons

**Figure 8:**
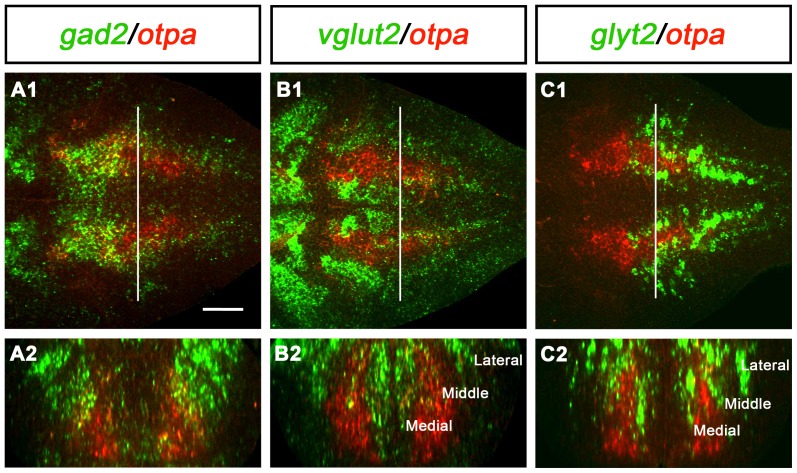
Expression of *otpa* in relation to gabaergic, glutamatergic and glycinergic markers in the hindbrain of wildtype larvae. Potential coexpression of *otpa* with gabaergic (*gad2*, A), glutamatergic (*vglut2*, B) and glycinergic (*glyt2*, C) markers was analyzed by double whole mount FISH at 3 dpf. A1, B1, and C1 are single plane dorsal views of the hindbrain, anterior is to the left. A2, B2, and C2 are cross-sections at the level of the hindbrain indicated by the white line in A1, B1, and C1, respectively. The orthogonal view cross sections were obtained from dorsal confocal stacks using the TransformJ Turn plugin of the ImageJ software. Scale bar is 50 µm.

**Figure 9:**
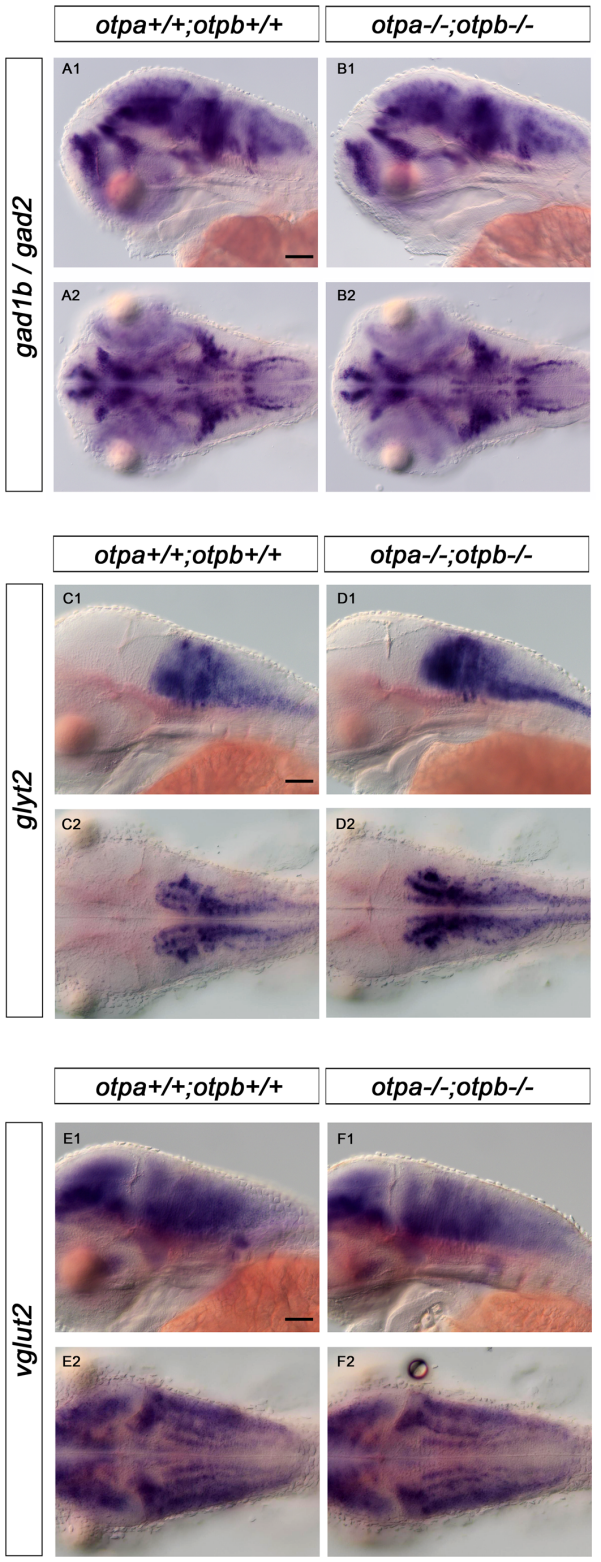
Expression of *gad1b/2, glyt2* and *vglut2* in wildtype and in *otp;otpb* double mutant larvae. Whole-mount in situ hybridization reveals no changes in expression of gabaergic (*gad1b/gad2), glycinergic (glyt2)and glutamatergic (vglut2)* in the hindbrain of *otpa;otpb* double mutant larvae at 3 dpf. (A1, B1, C1, D1, E1, F1) lateral view, dorsal up; (A2, B2, C2, D2, E2,F2) dorsal view, anterior at left. Scale bar is 50 µm.

Given that both *otpa* and *otpb* are expressed broadly in the hindbrain [8], [47], we analyzed potential effects of the loss of Otp function on the development of other neuronal populations in this region. Given the longitudinal extent of the *otp* expression domains in the hindbrain, we expected that *otp* may not be regulated in rhombomeric patterns, but correlate with some of the rostrocaudal hindbrain neurotransmitter stripes previously characterized in zebrafish [32], [48]. These publications distinguished three longitudinal stripes (medial, middle and lateral) on each side for glutamatergic and glycinergic neurons. We therefore analyzed coexpression of markers for gabaergic, glycinergic, and glutamatergic neurons with *otpa* expression in the hindbrain (Figure 8). There was no one-to-one correlation of *otpa* expression with any of these three transmitter types in the hindbrain. However, it appeared that a significant portion of the *otpa* expressing cells in the rostral hindbrain expressed gabaergic markers (Figure 8A). In contrast, in the medial and caudal hindbrain, only the lateral portion of the longitudinal *otpa* expression domain may be gabaergic. The *otpa* expression domain is limited medially and overlaps with the medial glutamatergic stripe, and appears to contain the middle glutamatergic stripe (Figure 8B). Similarly, coexpression of *otpa* and *glyt2* was detected in parts of the medial and middle glycinergic stripe (Figure 8C). To investigate whether loss of Otp activity affects any of these transmitter stripes, we analyzed the development of gabaergic (expression of *gad1b/gad2*), glycinergic (expression of *glyt2*) and glutamatergic (expression of *vglut2*) neurons in *otpa;otpb* double mutants. Surprisingly, we could not detect any significant differences in neurotransmitter-specific expression domains when *otpa;otpb* double mutants were compared to wildtype (Figure 9). However, these findings do not exclude that Otp may affect other aspects of differentiation of these neurons. These observations also suggest that the requirement for Otp activity during specification of *sst1.1*-expressing cells in the hindbrain is a specific function of Otp proteins, and is not caused by more global potential patterning defects of the hindbrain in *otpa;otpb* double mutants. Other potential roles of the broad Otp expression in the hindbrain still remain to be elucidated.

## Discussion

Neuroendocrine and neuromodulatory systems of the hypothalamus, including the DA systems, are central to the control of basic behavior patterns and physiology, but molecular mechanisms controlling their neuronal differentiation are not well understood. Otp is a transcription factor crucial for development of several neuronal types in the hypothalamus and preoptic region [2], [3], [8], [25], [47]. While zebrafish are an excellent model to study neuronal development, a genome duplication at the base of teleost evolution resulted in two paralogous copies of many genes [49], including *otp*. Here we have genetically dissected the contributions of the paralogous genes *otpa* and *otpb* to the specification of neuroendocrine and DA neuron types in the zebrafish larval brain. The analysis of the *otpb* single mutant and *otpa;otpb* double mutant phenotype has only now become possible through a potential null allele for the *otpb* gene in zebrafish isolated by the Sanger Zebrafish Mutation Project. This mutation causes a truncated protein that completely lacks the homeodomain. While both single *otpa* and *otpb* mutants are viable and develop into fertile adults, with no abnormal morphological phenotype, *otpa;otpb* double mutant embryos die at larval stages without any obvious morphological defect (data not shown).

We analyzed the role of Otp during the development of *th*, *crh*, *oxt*, *avp*, *trh* and *sst1.1-*expressing cells. In zebrafish both *otp* paralogous genes act partially redundant to accomplish a function equivalent to the single *OTP* gene in mice. Our experiments with *otpa*;*otpb* double mutants regarding DA neurons are in agreement with this view. Double mutants display a complete loss of DA groups 2, 4, 5 and 6 in the ventral diencephalon which is similar to what is observed in the *Otp−/−* mice, which lack all the neurons belonging to the A11 group [8].

For DA neuron specification in the posterior tubercular region *otpa* appears to provide most of Otp activity, since *otpa* mutants have a drastically reduced number of DA neurons, whereas *otpb* mutants do not. It has been previously reported that knocking down *otpb* alone by morpholino approach leads to a strong reduction of DA neurons in the ventral diencephalon [35], [47]. However, our analysis of the *otpb^sa0115^* mutant allele and our previous study using an *otpb* specific Morpholino [8] did not reveal a significant DA phenotype. We compared the sequences of the *otpb* Morpholino used in two publications [25], [47] with *otpa* and *otpb* sequences, and found that the *otpb* morpholino used by Del Giacco et al. (2006) may bind with only three mismatches also to *otpa*, while the *otpb* morpholino used by Eaton et al. (2006) may bind with four mismatches also to *otpa*. Thus, the results reported in these manuscripts for *otpb* knockdown may in part be attributed to the *otpb* morpholino used in these studies binding also to the *otpa* paralog. Our data contradicts the previously published hypothesis that *otpb* would be more relevant than *otpa* for diencephalic neurodifferentiation [50]. The different results may arise by non-specific effects of morpholino injections to knock down *otpb* gene function. Many of the common problems using morpholinos were reviewed in detail recently [51] and our results once more emphasize the need for a careful experimental setup when making conclusions from morpholino experiments, especially when addressing the function of paralogous genes during zebrafish development. Fortunately, gene-specific mutations are now more readily available through TILLING screens [52] and TALEN site specific mutagenesis [53].

A previous study has also reported RT-PCR data indicating that *otpb* may be expressed maternally and *otpb* mRNA deposited in to the egg [47]. This would raise the possibility that maternal *otpb* message may attenuate the zygotic *otpb* mutant phenotype. However, there are three lines of evidence suggesting that there is no significant maternal contribution of *otpb*: (1) systematic microarray analysis of expression mRNA profiles from zygote to late gastrula stages demonstrate that there is no specific *otpb* mRNA signal at blastula or gastrula stages [54]; (2) we have not been able to detect *otpb* message by whole mount *in situ* hybridization (unpublished data); (3) Del Giacco et al. (2006) also reveal in their manuscript that they were not able to detect *otpb* mRNA by WISH before the 3-somite stage [47]. We therefore conclude that if any maternal *otpb* mRNA persists beyond zygote stage, the amount is so low that it likely does not affect the phenotype. We further investigated the possibility that *otpa* and *otpb* may mutually contribute to regulation of their expression by analyzing *otpa* expression in *otpb* mutants and *otpb* expression in *otpa* mutants (Figure S4). We could not detect any influence of loss-of-function in one *otp* paralog on expression of the other paralog.

**Figure 10:**
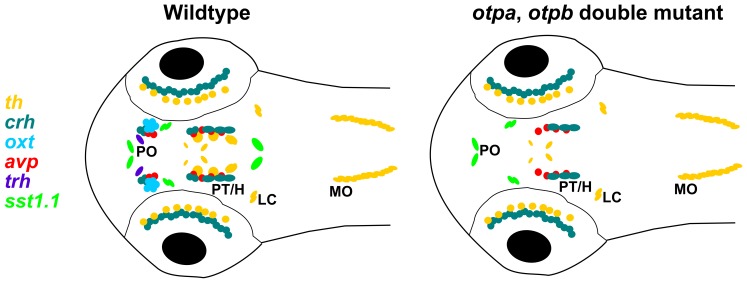
Schematic representation of neuroendocrine and dopaminergic cell groups affected in *otpa/otpb* double mutant larval zebrafish at 3 dpf. Schematic diagram showing the expression of the *th, sst1.1, trh, oxt, avp,* and *crh* neuronal groups analyzed in this study at 3 dpf in zebrafish larvae. For simplicity, the schematic representation does not include the entire expression patterns of all these 7 genes, but focuses on anatomical regions with Otp expression. The comparison of wildtype and *otpa;otpb* double mutant larvae reveals groups dependent on Otp activity. H, hypothalamus; LC, locus coeruleus; MO, medulla oblongata; PO, preoptic region; PT, posterior tuberculum.

Our analysis of *otpa*;*otpb* double mutant embryos clearly demonstrates the requirement for Otp activity by defined subsets of *crh*, *avp, trh* and *sst1.1* neuroendocrine cells in the posterior tubercular/hypothalamic, hindbrain as well as preoptic regions, and for essentially all preoptic *oxt* neuroendocrine cells at zebrafish larval stages. These findings are summarized schematically in Figure 10. For the specification of *crh*, *oxt* and *avp* neuroendocrine cells both *otp* paralogous genes appear to act mutually redundant, as no significant reduction in the number of cells is detectable in *otpa* or *otpb* single mutant embryos. However, ectopic *oxt*-expressing cells are apparent in *otpa* mutant embryos. The observation of ectopic *oxt*-expressing cells in *otpa* mutants resembles the phenotype caused by *sim1a* Morpholino knockdown [24]. This paper also reports a reduction of *oxt, avp* and *trh* expression in the PO region in *arnt2^m1055^* mutant and *sim1a* morpholino knockdown embryos [24]. *trh* expressing cells in the PO region are also reduced in *otpa* single mutants, but not in *otpb* single mutants.

Our findings that several neuroendocrine populations are completely lost in the PO of *otpa;otpb* double mutants is similar to what has been reported for *Otp−/−* mice. Otp in mice is expressed in the paraventricular (PVN), supraoptic (SON), anterior periventricular (aPV), and arcuate (ARN) nuclei, as well as in other parts of the central nervous system [2], [3]. In *Otp−/−* mice, TRH and CRH expression is completely lost in the PVN. AVP and OT (also known as OXT) expression is completely lost in the SON. In *Otp−/−* mice SS (also known as SST) has been shown to be absent in the aPV and in the ARN. In contrast, we could not detect a reduction in the number of *sst1.1* positive cells in the PO of *otp* mutants at 3 dpf. However, we have identified a group of *sst1.1* expressing neurons in the rostral hindbrain that depend on Otp activity in zebrafish embryos. Otp has been shown to be strongly expressed in the hindbrain in mice [3], but so far no abnormalities in hindbrain expression of SS have been reported for *Otp−/−* mice.

What may cause the slightly different effects of *otpa* and *otpb* on different neuroendocrine and DA populations? Hypothetically, this could be differences in Otpa and Otpb protein function, or differences in spatial and temporal expression patterns of both paralogous genes. Given the high conservation of Otpa and Otpb proteins (Fig. 1) as well as the observed partial functional redundancies, we favor the second option as cause for the differences. *otpa* and *otpb* have similar but in some regions slightly spatially shifted expression domains (Figure 2) [8], making different neuronal populations differentially sensitive to reduction of *otpa* and *otpb* activity.

While modulation of Otp activity levels in single mutant or transheterozygous *otpa* and *otpb* mutations has little effect on *crh* expression, Otp may have functions in behavioral physiology not detected in normal developmental assays. In a recent study an antibody against CRH protein was used to evaluate CRH expression, and under standard conditions no phenotype was detected in *otpa* mutants versus wild-type during development [55]. However, in the presence of a stressor stimulus, *crh* transcription was significantly induced in wildtype, but not in *otpa^m866^* mutant larvae. Similarly, the same study showed that transgene driven enhanced Otp expression caused increased *crh* transcription, suggesting that Otp may have a role in physiological stress related control of *crh* expression.

Given the complexity of neuronal phenotypes in the hypothalamus and preoptic region, additional types of neurons likely depend on Otp activity in this region. In a recent report, we could show that *opn4a* expressing cells in a specific cluster in the preoptic region are reduced in *otpa* mutants and absent in *otpa* and *otpb* double mutants [56]. This was surprising, as *opn4a* cells are supposed to be light sensing, adding them to the repertoire of neurosecretory and neuromodulatory neurons specified by Otp. A similarly interesting finding in our current study is the dependence of a large group of *sst1.1* expressing neurons in the rostral hindbrain on Otp activity, which provides the first evidence that the strong expression domains of *otpa* and *otpb* are indeed involved in neuronal differentiation in the hindbrain of vertebrates. It is likely that future studies on *otpa* and *otpb* function in zebrafish will identify additional neuronal groups and potentially neuronal circuits depending on Otp activity.

## Supporting Information

Figure S1 **Expression of** ***oxt, avp, trh, crh and sst1.1*** **in** ***otpa*** **and** ***otpb*** **mutant larvae.** Whole-mount in situ hybridization of 3 dpf larvae reveals changes of *oxt, avp, trh and crh* expression in the preoptic region (arrowhead in A, C, E, G) and reduction of *sst1.1* expression (arrowhead in I) in the hindbrain of *otpa−/−* mutant, *otpb+/−* heterozygous larvae. In contrast, no obvious change is detected in the preoptic region (arrowheads in B, D, F, H) and hindbrain (J) of *otpb−/−* mutant, *otpa+/−* heterozygous larvae. Dorsal view, anterior at left. Scale bar is 50 µm. H, hypothalamus; PO, preoptic region; PT, posterior tuberculum.
(TIF)

Figure S2 **Quantification of** ***oxt, avp, trh, crh and sst1.1*** **cell numbers in** ***otp*** **mutants.** Histogram illustrating the average number of *oxt* (A), *oxt* ectopic cells (B), *avp* (C), *trh* (D), *crh* preoptic region (E), *crh* anterior posterior tuberculum (F) and *sst1.1* rostral hindbrain (G) neurons. Y-axis gives number of stained neurons per embryo and anatomical group. Numbers in histogram bars provide the number of embryos imaged and analyzed. To evaluate differences for statistical significance, cell numbers from the different genotypes analyzed were compared with wildtype larvae using the Wilcoxon–Mann–Whitney rank-sum test. * P<0.05 one-tailed, ** P<0.01 one-tailed. Error bars indicate standard error of the mean.
(TIF)

Figure S3 **High-resolution imaging of neuroendocrine cells for cell counting.** Example of 3 dpf wildtype embryos imaged at single-cell resolution for quantification of cell numbers. (A) *oxt*, (B), *avp*, (C) *trh*, (D) *crh* expression analysis by WISH. For this figure, from the whole image stack with images at 1 µm spacing, sub-stacks of planes were used to generate a series of dorso-ventral Z-projections containing the region of interest. A higher magnification of regions of interest is shown on the right panel. Dorsal view, anterior at left. Scale bar is 100 µm. H, hypothalamus; HB, Hindbrain; PO, preoptic region; PT, posterior tuberculum.
(TIF)

Figure S4 **Expression of** ***otp*** **genes is not altered in** ***otp*** **mutant larvae.** Whole-mount in situ hybridization of 3 dpf larvae reveals no obvious changes of *otpb* expression in the preoptic region (A1, B1) and hindbrain (A2, B2) in *otpa* mutants. Similarly, no obvious changes in *otpa* expression were detected in the preoptic region (C1, D1) and hindbrain (C2, D2) expression domains in 3 dpf *otpb* mutants. Scale bar is 100 µm.
(TIF)
