# Supplementary figures and images for: Orthopedia Transcription Factor otpa and otpb Paralogous Genes Function during Dopaminergic and Neuroendocrine Cell Specification in Larval Zebrafish
Source: PLoS One. 2013 Sep 20;8(9):e75002. doi: 10.1371/journal.pone.0075002 (PMC3779234; doi:10.1371/journal.pone.0075002)

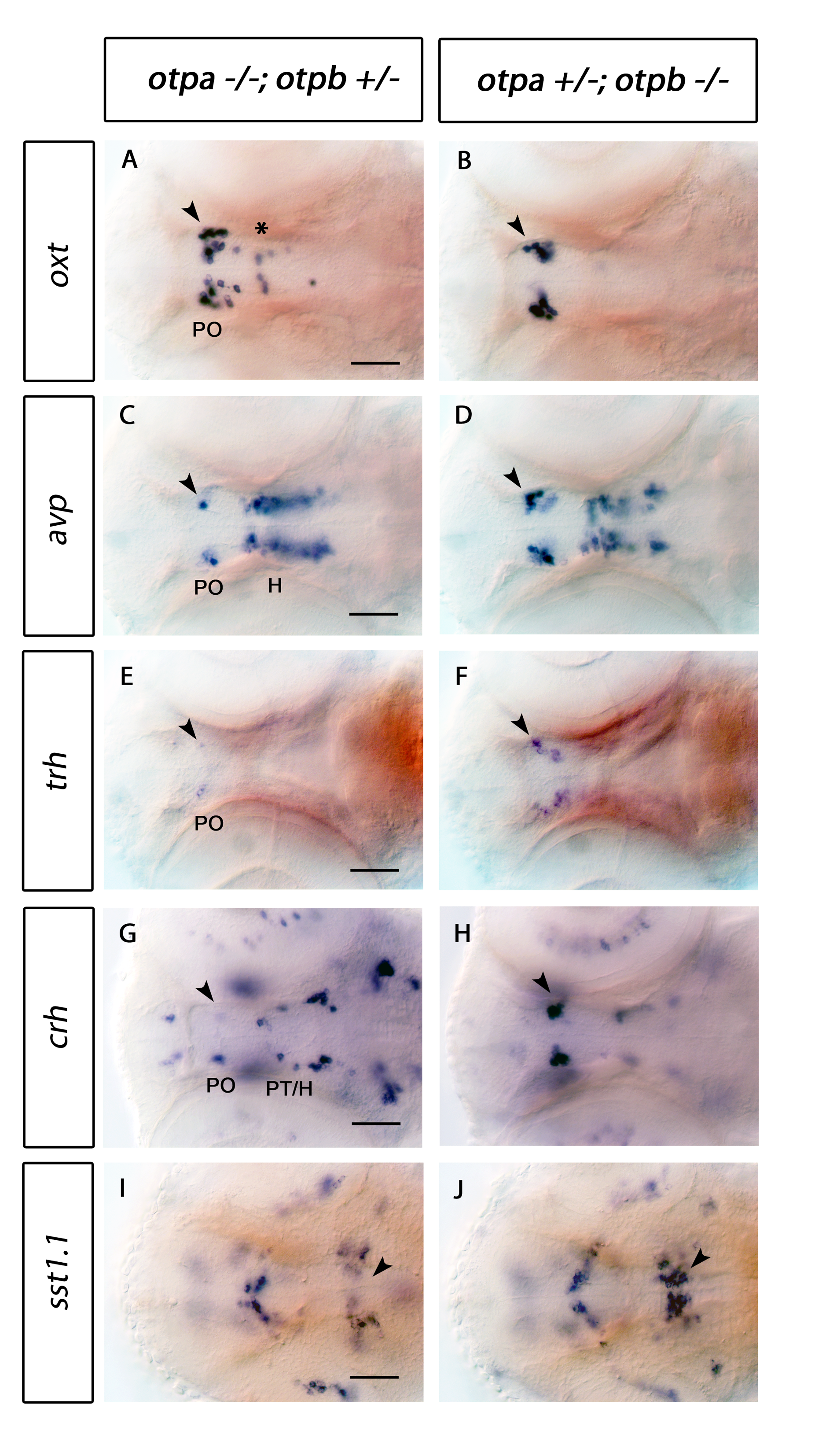

Supplement: Figure S1 — Expression of oxt, avp, trh, crh and sst1.1 in otpa and otpb mutant larvae. Whole-mount in situ hybridization of 3 dpf larvae reveals changes of oxt, avp, trh and crh expression in the preoptic region (arrowhead in A, C, E, G) and reduction of sst1.1 expression (arrowhead in I) in the hindbrain of otpa−/− mutant, otpb+/− heterozygous larvae. In contrast, no obvious change is detected in the preoptic region (arrowheads in B, D, F, H) and hindbrain (J) of otpb−/− mutant, otpa+/− heterozygous larvae. Dorsal view, anterior at left. Scale bar is 50 µm. H, hypothalamus; PO, preoptic region; PT, posterior tuberculum. (TIF) [file pone.0075002.s001.tif]

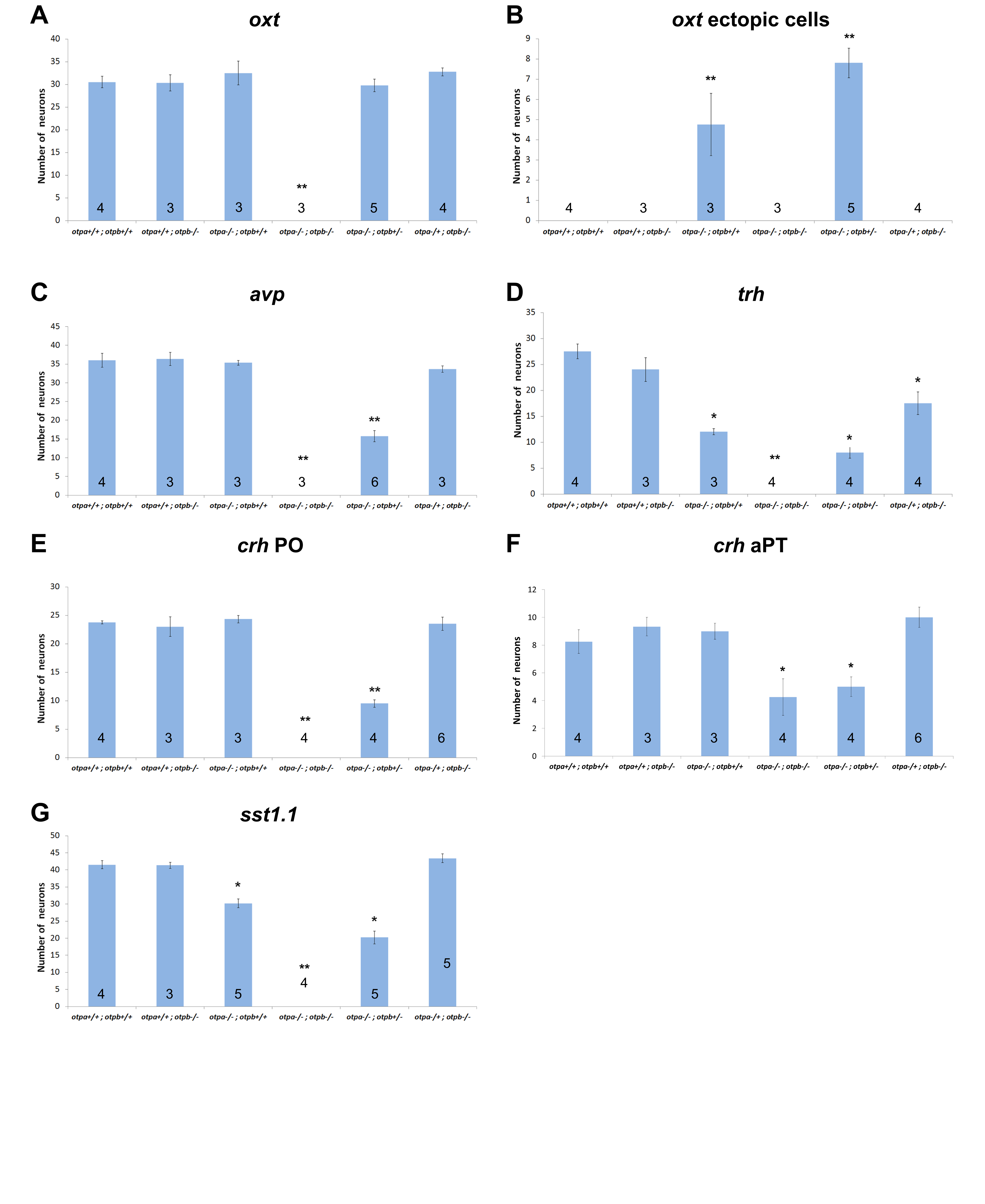

Supplement: Figure S2 — Quantification of oxt, avp, trh, crh and sst1.1 cell numbers in otp mutants. Histogram illustrating the average number of oxt (A), oxt ectopic cells (B), avp (C), trh (D), crh preoptic region (E), crh anterior posterior tuberculum (F) and sst1.1 rostral hindbrain (G) neurons. Y-axis gives number of stained neurons per embryo and anatomical group. Numbers in histogram bars provide the number of embryos imaged and analyzed. To evaluate differences for statistical significance, cell numbers from the different genotypes analyzed were compared with wildtype larvae using the Wilcoxon–Mann–Whitney rank-sum test. * P<0.05 one-tailed, ** P<0.01 one-tailed. Error bars indicate standard error of the mean. (TIF) [file pone.0075002.s002.tif]

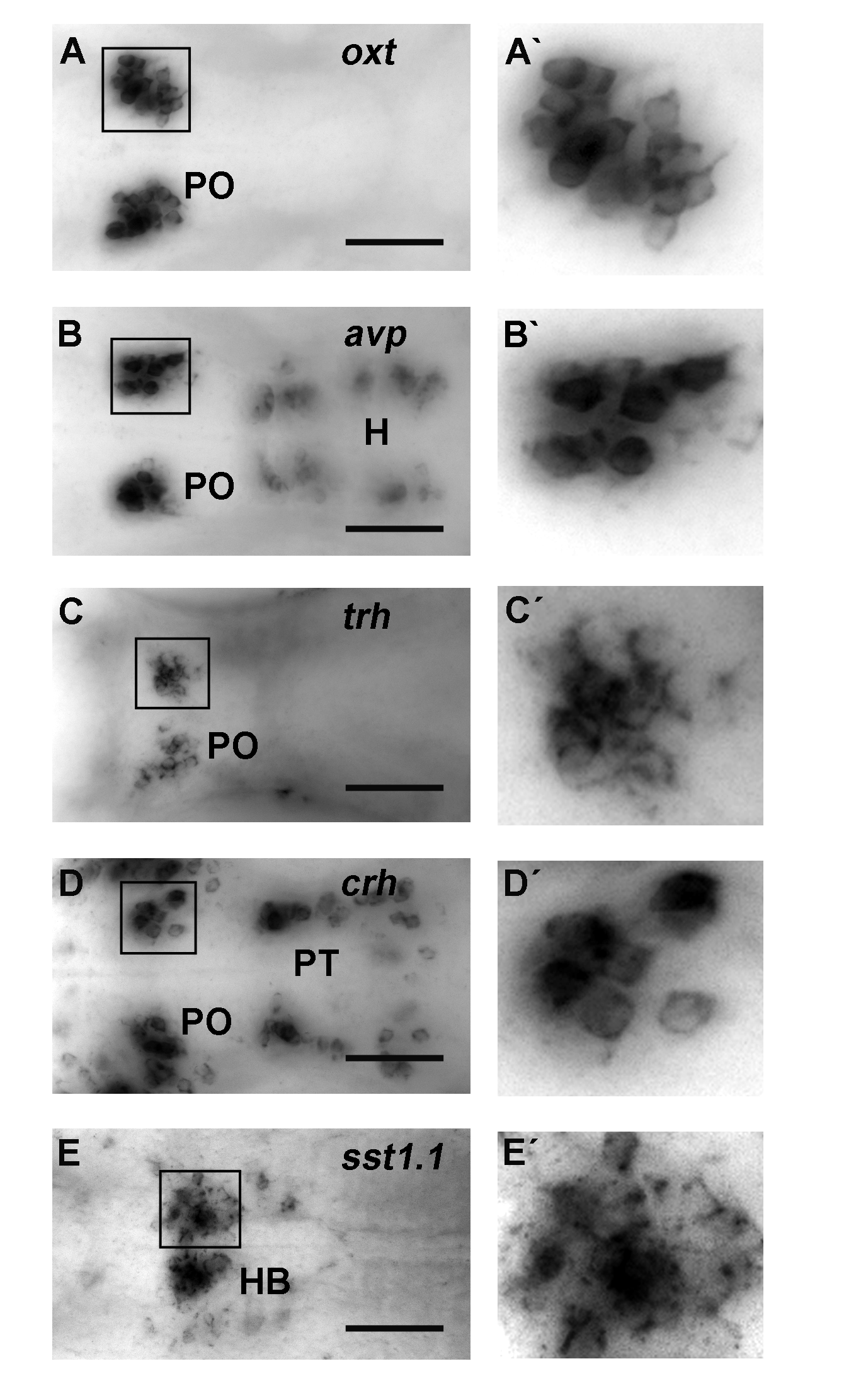

Supplement: Figure S3 — High-resolution imaging of neuroendocrine cells for cell counting. Example of 3 dpf wildtype embryos imaged at single-cell resolution for quantification of cell numbers. (A) oxt, (B), avp, (C) trh, (D) crh expression analysis by WISH. For this figure, from the whole image stack with images at 1 µm spacing, sub-stacks of planes were used to generate a series of dorso-ventral Z-projections containing the region of interest. A higher magnification of regions of interest is shown on the right panel. Dorsal view, anterior at left. Scale bar is 100 µm. H, hypothalamus; HB, Hindbrain; PO, preoptic region; PT, posterior tuberculum. (TIF) [file pone.0075002.s003.tif]

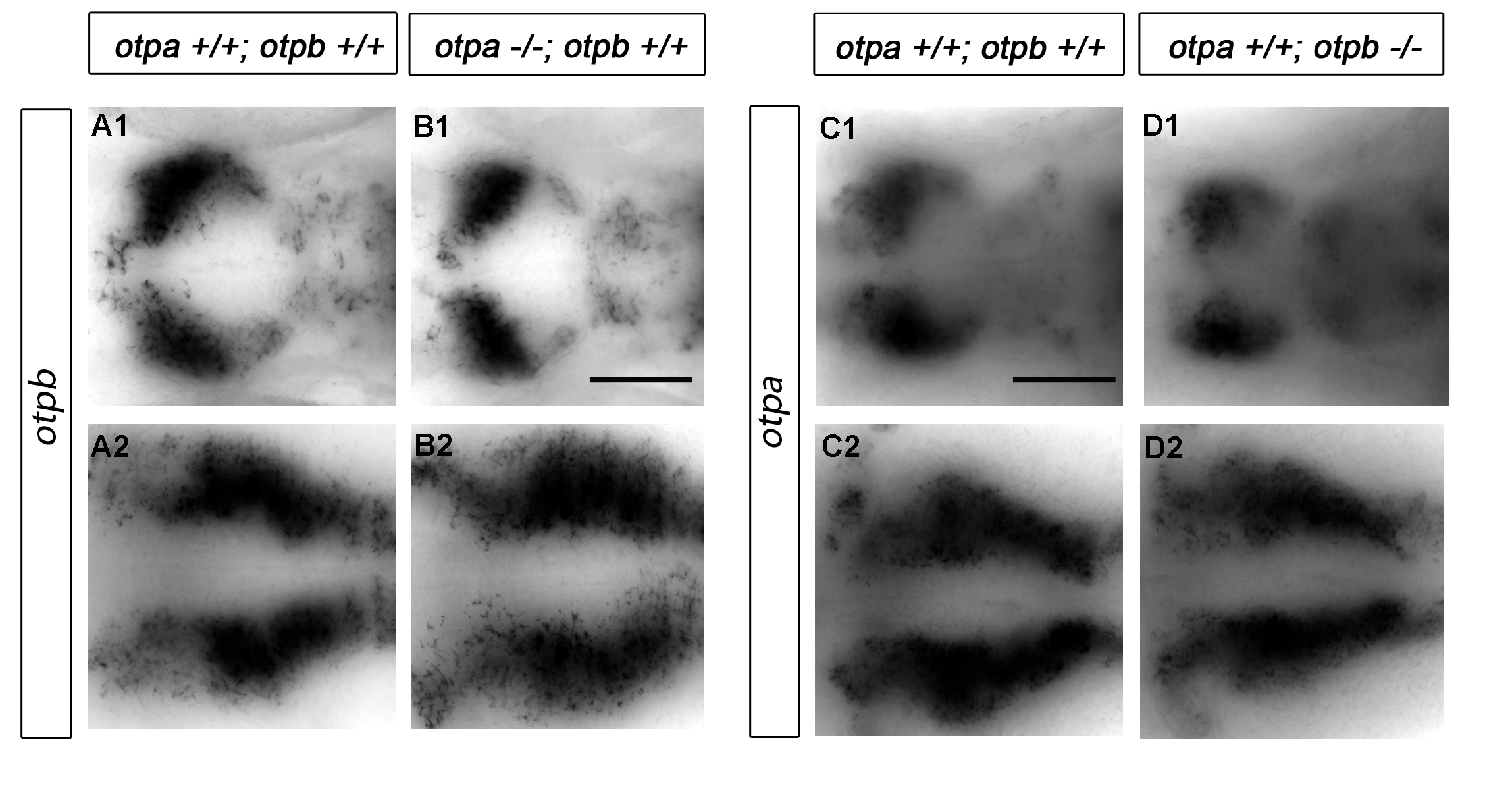

Supplement: Figure S4 — Expression of otp genes is not altered in otp mutant larvae. Whole-mount in situ hybridization of 3 dpf larvae reveals no obvious changes of otpb expression in the preoptic region (A1, B1) and hindbrain (A2, B2) in otpa mutants. Similarly, no obvious changes in otpa expression were detected in the preoptic region (C1, D1) and hindbrain (C2, D2) expression domains in 3 dpf otpb mutants. Scale bar is 100 µm. (TIF) [file pone.0075002.s004.tif]
